# Left ventricular-arterial coupling as a predictor of stroke volume response to norepinephrine in septic shock – a prospective cohort study

**DOI:** 10.1186/s12871-021-01276-y

**Published:** 2021-02-17

**Authors:** Xiaoyang Zhou, Jianneng Pan, Yang Wang, Hua Wang, Zhaojun Xu, Weibo Zhuo

**Affiliations:** 1 Department of Intensive Care Medicine, HwaMei Hospital, University of Chinese Academy of Sciences, Ningbo, Zhejiang, 315000 China; 2 Ningbo Institute of Life and Health Industry, University of Chinese Academy of Sciences, Ningbo, Zhejiang, 315000 China; 3 Department of Intensive Care Medicine, Ningbo Fenghua District Hospital of Traditional Chinese Medicine Medical Community, Ningbo, Zhejiang, 315500 China

**Keywords:** Septic shock, Stroke volume, Norepinephrine, Cardiovascular, Ventricular-arterial coupling

## Abstract

**Background:**

Left ventricular-arterial coupling (VAC), defined as the ratio of arterial elastance (Ea) to left ventricular end-systolic elastance (Ees), is a key determinant of cardiovascular performance. This study aims to evaluate whether left VAC can predict stroke volume (SV) response to norepinephrine (NE) in septic shock patients.

**Methods:**

This was a prospective cohort study conducted in an intensive care unit of a tertiary teaching hospital in China. We recruited septic shock patients who had persistent hypotension despite fluid resuscitation and required NE to maintain mean arterial pressure (MAP) > 65 mmHg. Those patients in whom the target MAP was not reached after NE infusion were ineligible. Echocardiographic variables were measured before (baseline) and after NE infusion. SV responder was defined by a ≥ 15% increase in SV after NE infusion.

**Results:**

Of 34 septic shock patients included, 19 (56%) were SV responders. Before NE infusion, SV responders had a lower Ees (1.13 ± 0.24 mmHg/mL versus 1.50 ± 0.46 mmHg/mL, *P* = 0.005) and a higher Ea/Ees ratio (1.47 ± 0.40 versus 1.02 ± 0.30, *P* = 0.001) than non-responders, and Ea in SV responders was comparable to that in non-responders (1.62 ± 0.36 mmHg/mL versus 1.43 ± 0.28 mmHg/mL, *P* = 0.092). NE significantly increased Ea and Ees in both groups. The Ea/Ees ratio was normalized by NE administration in SV responders but unchanged in non-responders. The baseline Ea/Ees ratio was positively correlated with NE-induced SV increases (r = 0.688, *P* < 0.001). Logistic regression analysis indicated that the baseline Ea/Ees ratio was a predictor of SV increases induced by NE (odd ratio 0.008, 95% confidence interval (CI): 0.000 to 0.293), with an area under the receiver operating characteristic curve of 0.816 (95% CI: 0.646 to 0.927).

**Conclusions:**

The left VAC has the ability to predict SV response to NE infusion in septic shock patients.

**Trial registration:**

Chinese Clinical Trial Registry, ChiCTR1900024031, Registered 23 June 2019 - Retrospectively registered, http://www.chictr.org.cn/edit.aspx?pid=40359&htm=4.

## Background

Currently, septic shock remains the leading cause of death in the intensive care unit (ICU) with a high mortality of around 38% [1]. Fluid administration is a very important treatment for septic shock, but it is always accompanied by an increased risk of fluid overload and seems to be insufficient to restore the arterial pressure due to the depressed vasomotor tone. Thus, vasopressor is advocated to be applied early to achieve a minimum acceptable arterial pressure to guarantee organ perfusion [2–4].

Norepinephrine (NE) is recommended as the first choice of vasopressor in the management of septic shock [5]. As a potent α1-adrenergic agent with β1-adrenergic properties, NE can increase the left ventricular afterload and myocardial oxygen consumption through restoring vasomotor tone and subsequently improving arterial pressure [6, 7]. On the other hand, NE can improve cardiac contractility through stimulating β1-adrenergic receptors and improving coronary perfusion by increasing diastolic arterial pressure (DAP) [6], and it can also increase the left ventricular preload by redistributing venous blood from unstressed to stressed blood volume [2, 8, 9]. Given the wide spectrum of impacts of NE on cardiovascular performance, the overall cardiovascular effects of NE are difficult to determine.

It has been well described that the mechanical efficiency of the cardiovascular system depends on the interactions between the heart and the arterial system [10–12], namely left ventricular-arterial coupling (VAC), which is measured by the ratio of arterial elastance (Ea) to left ventricular end-systolic elastance (Ees). In the physiological conditions, the cardiac function is matched well with the arterial system, and this interaction is modulated dynamically to provide an optimal SV and arterial pressure to perfuse the organ and tissue [10]. However, this well-matched interaction will be inevitably broken in some pathological cases, such as septic shock [13], finally causing circulatory failure and worse prognosis [13–15]. Among interventions for the treatment of circulatory failure, the optimal treatment should be those that improve the work efficacy of the cardiovascular system with the lowest energetic consumption, which refers to high mechanical efficiency. Therefore, it is of interest to explore the effect of NE on the interactions between the heart and the arterial system, since NE exhibited complex effects on cardiovascular performance. Moreover, a description of the cardiovascular effects of NE will facilitate a better understanding of the pathophysiologic changes of hemodynamics during NE infusion. We therefore conducted this study to describe the relationship between the left VAC and the cardiovascular response to NE in septic shock patients. We hypothesized that the left VAC can predict SV response to NE in septic shock, given the fact that the left VAC determines the stroke volume (SV), left ventricular ejection fraction (LVEF), and ejection pressure [10, 16], and it possesses independent diagnostic and prognostic value in multiple diseases [17].

## Materials and methods

This was a prospective cohort study conducted between October 2018 and January 2020 in the 20-bed ICU of HwaMei Hospital, University of Chinese Academy of Sciences (Ningbo, China). This study was conducted in compliance with the Declaration of Helsinki and approved by the institutional ethics committee in our hospital (PJ-NBEY -KY-2019-014-01) and adhered to the Strengthening the Reporting of Observational Studies in Epidemiology (STROBE) guidelines. Written informed consent was obtained from the patients or their next of kin. This study was part of a study program that was registered in the Chinese Clinical Trial Registry (ChiCTR1900024031).

### Patients

Adult patients (age > 18 years) with septic shock, who had persistent hypotension despite fluid resuscitation and required NE to maintain mean arterial pressure (MAP) > 65 mmHg, were considered for enrollment after ICU admission. Septic shock was diagnosed according to the criteria of the third international consensus definitions for sepsis and septic shock [18]. The exclusion criteria included: 1) Refractory shock patients in whom the target MAP was not reached after NE infusion and needed to infuse other vasopressors or inotropic agents to maintain MAP; 2) Patients with atrial fibrillation; 3) Patients who were receiving vasoactive agents or cardiac function assist device (such as pacemaker) at the time of enrollment; 4) Patients who had poor echogenicity or could not tolerate the transthoracic echocardiography (TTE) examination.

### Study protocol

Radial artery catheterization was performed in all patients after their ICU admission to measure the invasive arterial pressure. The initial resuscitation practice adhered to the recommendations of the Surviving Sepsis Campaign [5] and its update [4]. These practices included fluid resuscitation, appropriate antibiotic therapy, source control, vasoactive medications, and organ support. Fluid responsiveness was evaluated using dynamic echocardiographic indices (e.g. the respiratory variation in inferior vena cava diameter, the passive leg raising-induced changes in SV) before NE infusion start. Whether start NE infusion was decided by the physician in charge based on the MAP, fluid non-responsiveness, and fluid volume administered in each patient (at least 30 mL/kg of crystalloid fluid within the first 3 h). NE dose was adjusted to reach the target MAP (more than 65 mmHg) and maintain MAP stabilization. MAP stabilization was defined as a variation of MAP < 10% with NE infusion during a period of at least 15 min [19]. Other vasoactive drugs or inotropic agents were not considered before the end of the study period. Additional sedative and analgesic drugs were used to facilitate invasive mechanical ventilation (IMV) in patients treated with IMV. Modifications of ventilator setting or dose of sedative and analgesic drugs and fluid challenges were not allowed during the study period.

### Data collection

We recorded the demographic information, source of infection, causative pathogen in culture, and concomitant disease for all patients at ICU admission. The blood gas, acute physiology and chronic health evaluation (APACHE) II score, and sequential organ failure assessment (SOFA) score at the time of enrolment were also collected for each patient. Central venous pressure (CVP) was measured before and after NE infusion for all subjects. The ratio of arterial oxygen partial pressure (PaO_2_) to fractional inspired oxygen (FiO_2_), ventilator parameters, type of sedative and analgesic drug, and length of IMV were collected for patients treated with IMV. Finally, we recorded and analyzed the dose of NE administered, urine output, the time elapsed from NE infusion start to MAP stabilization, duration of ICU stay, and cumulative fluid volume (before NE infusion, during the study period, and within the first 24 h after septic shock diagnosis). All patients were followed up to hospital discharge.

### Transthoracic echocardiography

TTE examination was performed for all patients by an independent ICU physician using a Philips CX50 ultrasound system (Philips Medical System, Suresnes, France). This trained operator had an operating experience in TTE for more than 3 years and was blinded to our study protocol. The left lateral decubitus position was preferred to obtain a good cardiac ultrasound image. All patients were connected to an electrocardiogram.

In the apical four-chamber view, left ventricular end-diastolic volume (LVEDV) and left ventricular end-systolic volume (LVESV) were measured using Simpson’s method, then LVEF was calculated. Continuous Doppler transaortic flow was obtained from the apical five-chamber view to measure the aortic velocity-time integral (VTI), pre-ejection time (T_pre-e_), and total systolic time (T_tot-s_). The diameter of the left ventricular outflow tract (LVOT) was measured in the parasternal long-axis view, and the area of LVOT was then calculated. Simultaneously, heart rate (HR), systolic arterial pressure (SAP), and DAP, as well as MAP, were also measured at the time of TTE examination. Finally, SV was calculated using the formula: SV = VTI × LVOT area, and cardiac output (CO) was calculated as SV × HR. Ea was estimated as (0.9 × SAP)/SV [20], and Ees was calculated using the single-beat method proposed by Chen et al. [21]. According to the previous publications [22, 23], Ea/Ees > 1.36 was considered as left ventricular-arterial uncoupling.

All measurements were performed at two time points: starting NE infusion (before NE infusion, baseline) and immediately after MAP stabilization (after NE infusion), regardless of the respiratory cycle. The representative value for each variable was estimated as the average value of three consecutive measurements. The NE-induced SV increase was employed to distinguish the SV responder (NE-induced SV increases ≥15%) from non-responder (NE-induced increases < 15%), where the NE-induced SV increase was calculated as (SV after NE infusion – SV before NE infusion)/ SV before NE infusion × 100%.

### Statistical analysis

The distribution of continuous variables was tested for normality using the Kolmogorov–Smirnov test. Normally distributed variables were expressed as mean ± standard deviation (SD), and variables with skewed distribution were presented as median and interquartile range (IQR). Categorical variables were expressed as frequency and percentages. Comparisons between SV responders and non-responders were assessed using the Student t test, Mann-Whitney U test, or Fisher exact test, as appropriate. Comparisons between the two time points within a group were assessed using the Student paired t test. The log-rank test was used to compare hospital mortality between the two groups. Pearson correlation coefficient was calculated to test the relationship between the baseline Ea/Ees ratio and other cardiovascular variables (including HR, SAP, CVP, LVEDV, LVEF, SV, and NE-induced SV increases) and to investigate whether NE-induced changes in Ea depend more on changes in SAP or SV. Univariate logistic regression analyses were used to screen the potential predictors of SV increase induced by NE. Given the small sample size, multivariate analysis was not performed. Receiver operating characteristic (ROC) curve was constructed for the Ea/Ees ratio, SV, SAP, and LVEDV at baseline to discriminate the SV responder from SV non-responder, and the optimal cutoff value was determined by the maximum of Youden index.

A sample size of 34 subjects was calculated to have a power of at least 90% to prove the hypothesis that the baseline Ea/Ees ratio could predict an increase in SV of ≥15% in response to NE with an area under the ROC curve (AUC) of 0.8, α of 0.05. The coefficient of variation (CV) and least significant change (LSC) were calculated to assess the intra-observer reproducibility for these directly measured ultrasound variables, including LVEDV, LVESV, VTI, T_pre-e_ and T_tot-s_, in 10 randomly selected patients. Two-sided *P* value < 0.05 was considered as statistical significance. Data analyses were performed using the statistical software SPSS 17.0 (IBM, New York, USA).

## Results

**Fig. 1:**
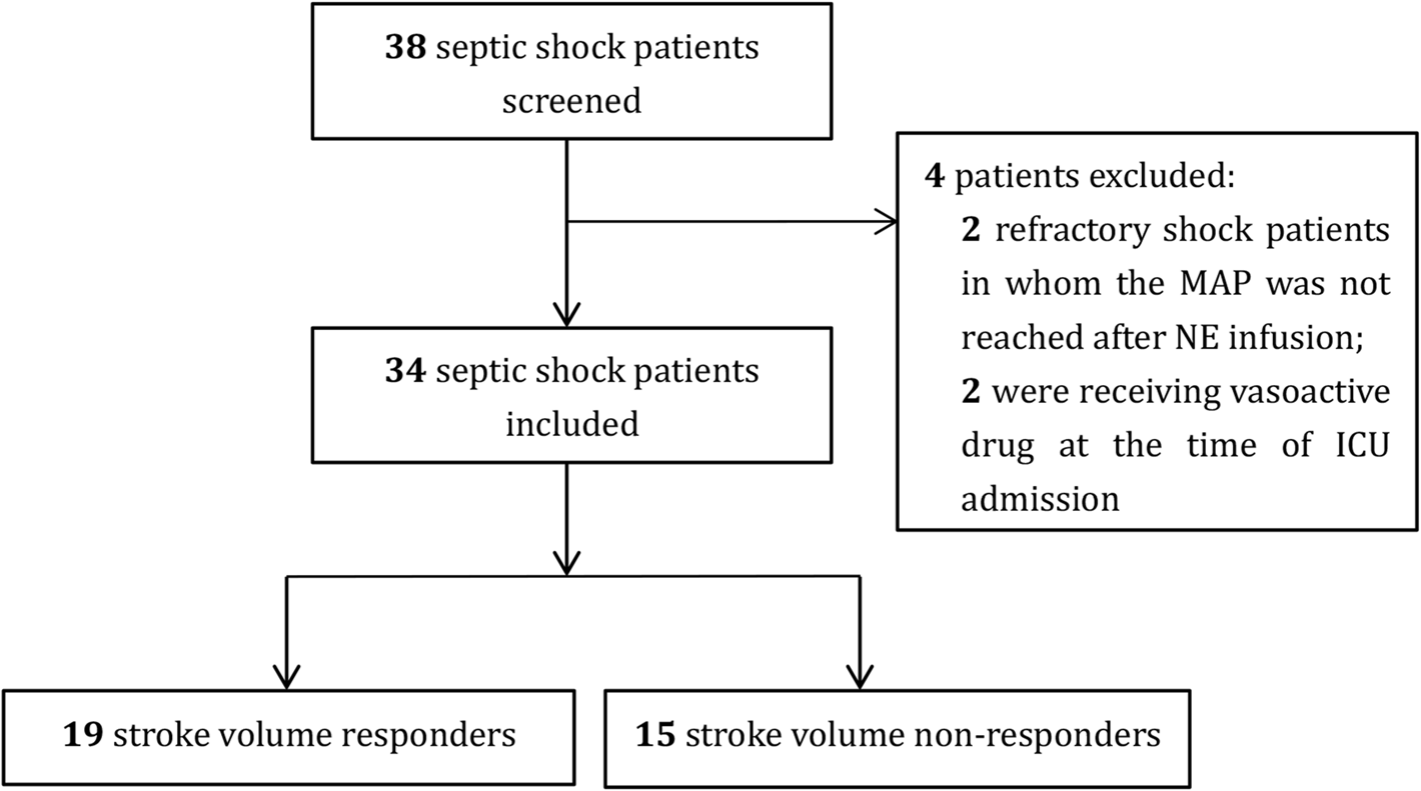
Flow chart of this study. NE norepinephrine; ICU intensive care unit

A total of 38 septic shock patients were initially consecutively screened for enrollment. After excluding 4 ineligible patients, we included 34 subjects, of which 19 were SV responders and 15 were SV non-responders (Fig. 1). The demographic characteristics of the included patients are summarized in details in Table 1. The baseline characteristics of responders were comparable to that of non-responders. Most of the included patients (71%) received IMV during the study period, and the duration of IMV was similar between groups. SV non-responders probably received more fluid during the first 24 h after the onset of septic shock than responders (*P* = 0.061). However, the cumulative fluid volumes before NE infusion and during the study period were similar between groups. The duration of ICU stay and in-hospital mortality did not differ between groups. In the whole studied population, the average value of Ea/Ees ratio before NE infusion was 1.27 ± 0.42, and 10 patients (29%) had an uncoupled ventricular-arterial interaction with an Ea/Ees ratio of > 1.36.

**Table 1:** Clinical characteristics and demographic data of the study participants

Variables	All patients (*n* = 34)	SV responders (*n* = 19)	SV non-responders (*n* = 15)	*P* value ^a^
Age (years, mean ± SD)	70 ± 12	69 ± 13	73 ± 11	0.368
Gender [Male, n (%)]	24 (71%)	14 (74%)	10 (67%)	0.718
Body mass index (kg/m^2^, mean ± SD)	22.5 ± 3.1	22.6 ± 3.1	22.5 ± 3.3	0.932
Body surface area (m^2^, mean ± SD)	1.65 ± 0.16	1.68 ± 0.17	1.62 ± 0.16	0.300
APACHE II score (mean ± SD)	20 ± 6	21 ± 5	20 ± 6	0.644
SOFA score (mean ± SD)	9 ± 3	9 ± 3	8 ± 2	0.554
Source of infection, n (%)				
Lung	18 (53%)	11 (58%)	7 (47%)	0.730
Urinary tract	7 (21%)	3 (16%)	4 (27%)	0.672
Abdomen	7 (21%)	5 (26%)	2 (13%)	0.426
Bloodstream	7 (21%)	3 (16%)	4 (27%)	0.672
Others	4 (12%)	2 (11%)	2 (13%)	1.000
Co-morbidities, n (%)				
Hypertension	16 (47%)	9 (47%)	7 (47%)	1.000
Diabetes	10 (29%)	7 (37%)	3 (20%)	0.451
Chronic obstructive pulmonary disease	3 (9%)	2 (11%)	1 (7%)	1.000
Coronary heart disease	2 (6%)	2 (11%)	0 (0%)	0.492
Chronic kidney disease	2 (6%)	1 (5%)	1 (7%)	1.000
Pathogen type in culture, n (%)				
Gram-negative	15 (44%)	9 (47%)	6 (40%)	0.624
Gram-positive	1 (3%)	1 (5%)	0 (0%)	
Mixed	2 (6%)	1 (5%)	1 (7%)	
Fungus	2 (6%)	0 (0%)	2 (13%)	
No pathogen	14 (41%)	8 (42%)	6 (40%)	
Patients receiving IMV, n (%)	24 (71%)	15 (79%)	9 (60%)	0.276
PaO_2_/FiO_2_ (mean ± SD)	262 ± 135	248 ± 134	285 ± 142	0.518
PEEP (cm H2O, mean ± SD)	6 ± 1	5 ± 1	6 ± 2	0.479
Tidal volume (mL/kg of predicted body weight, mean ± SD)	7.1 ± 1.3	6.8 ± 1.2	7.6 ± 1.3	0.129
Fentanyl, n (%)	15 (44%)	10 (53%)	5 (33%)	0.314
Midazolam, n (%)	15 (44%)	10 (53%)	5 (33%)	0.314
Propofol, n (%)	18 (53%)	11 (58%)	7 (47%)	0.730
Duration of IMV [days, median (IQR)]	6 (3–12)	6 (3–13)	5 (3–16)	0.719
Serum lactate level (mmol/L, mean ± SD)	3.5 ± 2.7	2.9 ± 1.4	4.2 ± 3.7	0.194
Patients with left ventricular-arterial uncoupling before NE infusion, n (%)	10 (29%)	8 (42%)	2 (13%)	0.128
Time from NE infusion start to MAP stabilization [min, median (IQR)]	95 (39–158)	86 (37–180)	100 (55–135)	0.862
Cumulative fluid volume before NE infusion (mL, mean ± SD)	1638 ± 569	1542 ± 523	1758 ± 620	0.278
Cumulative fluid volume during the study period [mL, median (IQR)]	193 (90–309)	180 (60–350)	210 (120–305)	0.490
Cumulative fluid volume within the first 24 h (mL, mean ± SD)	3744 ± 1251	3389 ± 1181	4194 ± 1228	0.061
NE dose (μg/kg/min, median (IQR))	0.254 (0.131–0.556)	0.22 (0.09–0.556)	0.44 (0.182–0.556)	0.167
Urine output (mL/kg/h, mean ± SD)	1.32 ± 0.73	1.13 ± 0.41	1.55 ± 0.97	0.133
Duration of ICU stay [days, median (IQR)]	7 (5–16)	7 (6–16)	12 (4–17)	0.958
In-hospital mortality, n (%)	9 (26%)	6 (32%)	3 (20%)	0.485

^*a*^ *P* value for comparisons of SV responders and SV non-responders

SV stroke volume; APACHE acute physiology and chronic health evaluation; SOFA sequential organ failure assessment; IMV invasive mechanical ventilation; PaO_2_ arterial oxygen partial pressure; FiO_2_ fractional inspired oxygen; PEEP positive end-expiratory pressure; NE norepinephrine; MAP mean arterial pressure; ICU intensive care unit; SD standard deviation; IQR interquartile range

### Intra-observer reproducibility

As shown in Table 2, the intra-observer reproducibility for the directly measured ultrasound variables was acceptable.

**Table 2:** Intra-observer reproducibility for directly measured ultrasound variables

Variables	LVEDV	LVESV	VTI	T_pre-e_	T_tot-s_
CV (%, 95 CI)	3.6 (2.8–4.5)	4.7 (3.7–5.7)	1.9 (1.3–2.5)	6.1 (3.9–8.4)	2.3 (1.7–2.9)
LSC (%, 95 CI)	5.8 (4.5–7.2)	7.6 (6.0–9.2)	3.0 (2.1–4.0)	9.8 (6.2–13.4)	3.6 (2.7–4.6)

CV coefficient of variation; LSC least significant change; LVEDV left ventricular end-diastolic volume; LDESV left ventricular end-systolic volume; VTI velocity-time integral; T_pre-e_ pre-ejection time; T_tot-s_ total systolic time; CI confidence interval

### Cardiovascular response to norepinephrine infusion

**Fig. 2:**
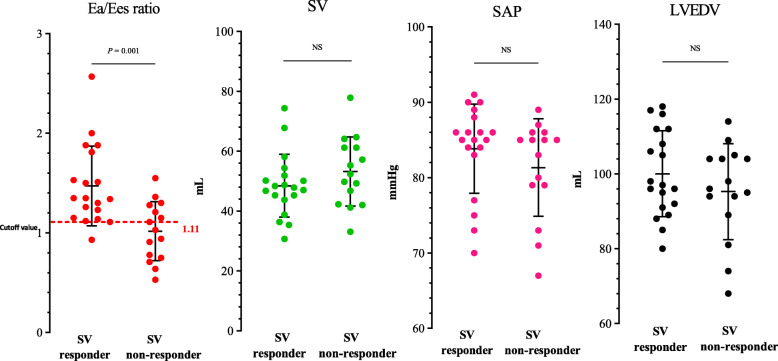
Scatter plot of individual cardiovascular variables at baseline. The solid line represents mean ± standard deviation, and the dotted line represents the optimal cutoff value. Ea effective arterial elastance; Ees left ventricular effective end-systolic elastance; SV stroke volume; SAP systolic arterial pressure; LVEDV left ventricular end-diastolic volume

Before NE infusion, SV responders had a lower VTI, lower LVEF, and higher LVESV than non-responders. The HR, SAP, DAP, MAP, CVP, SV, LVEDV, cardiac index, T_pre-e_, and T_tot-s_ in responders were comparable to that in non-responders (all *P* > 0.05). Although Ea did not differ between groups, Ees in responders was significantly lower than that in non-responders (*P* = 0.005), thus resulted in a higher Ea/Ees ratio in responders than non-responders (*P* = 0.001) (Table 3, Fig. 2).

**Table 3:** Cardiovascular responses to norepinephrine in stroke volume responders and non-responders

Variables	SV responders (n = 19)		SV non-responders (n = 15)		*P* value ^a^	*P* value ^b^
	Before NE	After NE	Before NE	After NE		
HR (beats/min)	112 ± 19	104 ± 20 ^c^	106 ± 18	96 ± 21 ^c^	0.384	0.255
SAP (mmHg)	84 ± 6	112 ± 14 ^d^	81 ± 6	109 ± 11 ^d^	0.247	0.456
DAP (mmHg)	48 ± 5	64 ± 9 ^d^	47 ± 5	56 ± 5 ^d^	0.535	0.006
MAP (mmHg)	60 ± 4	80 ± 9 ^d^	59 ± 4	73 ± 5 ^d^	0.269	0.027
CVP (mmHg)	9 ± 4	10 ± 3	8 ± 5	9 ± 3	0.498	0.329
VTI (cm)	16.9 ± 3.2	20.3 ± 3.3 ^d^	20.3 ± 4.4	21.4 ± 4.1 ^c^	0.012	0.380
SV (mL)	48 ± 10	58 ± 11 ^d^	53 ± 12	56 ± 11 ^c^	0.219	0.534
LVEDV (mL)	100 ± 12	104 ± 12 ^d^	95 ± 13	98 ± 12 ^c^	0.261	0.145
LVESV (mL)	52 ± 7	48 ± 6 ^d^	43 ± 10	43 ± 9	0.004	0.061
LVEF (%)	47 ± 6	54 ± 6 ^d^	54 ± 8	56 ± 8	0.006	0.255
Cardiac index (L/min/m^2^)	3.2 ± 0.9	3.6 ± 1.0 ^d^	3.5 ± 0.8	3.3 ± 0.8	0.440	0.276
T_pre-e_ (ms)	61 ± 17	59 ± 14	53 ± 17	56 ± 14	0.145	0.587
T_tot-s_ (ms)	227 ± 45	246 ± 44 ^c^	253 ± 40	280 ± 41 ^e^	0.083	0.029
Ea (mmHg/mL)	1.62 ± 0.36	1.79 ± 0.42 ^c^	1.43 ± 0.28	1.81 ± 0.40 ^d^	0.092	0.877
Ees (mmHg/mL)	1.13 ± 0.24	1.50 ± 0.39 ^d^	1.50 ± 0.46	1.82 ± 0.56 ^d^	0.005	0.057
Ea/Ees ratio	1.47 ± 0.40	1.24 ± 0.32 ^d^	1.02 ± 0.30	1.06 ± 0.34	0.001	0.145

The data are presented as mean ± standard deviation

^a^ *P* value for comparisons of SV responders and non-responders before NE infusion; ^b^ *P* value for comparisons of SV responders and non-responders after NE infusion; ^c^ *P* < 0.01, ^d^ *P* < 0.001, and ^e^ *P* < 0.05 for comparisons of before and after NE infusion within group

SV stroke volume; NE norepinephrine; HR heart rate; SAP systolic arterial pressure; DAP diastolic arterial pressure; MAP mean arterial pressure; CVP central venous pressure; VTI velocity-time integral; LVEDV left ventricular end-diastolic volume; LDESV left ventricular end-systolic volume; LVEF left ventricular ejection fraction; T_pre-e_ pre-ejection time; T_tot-s_ total systolic time; Ea effective arterial elastance; Ees left ventricular effective end-systolic elastance

**Fig. 3:**
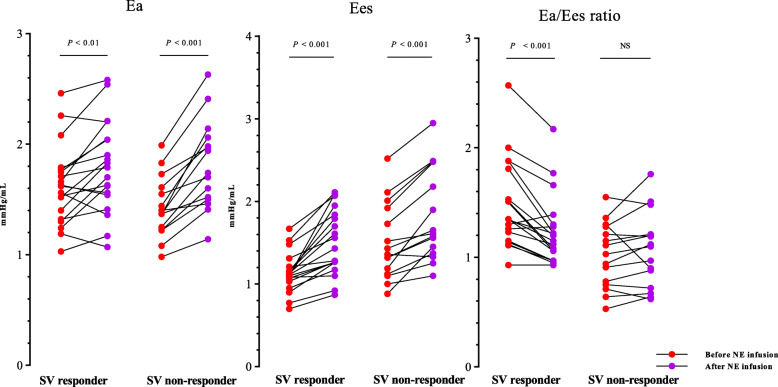
Individual changes in Ea, Ees, and Ea/Ees ratio after norepinephrine infusion. Ea effective arterial elastance; Ees left ventricular effective end-systolic elastance; SV stroke volume; NE norepinephrine

In both groups, NE significantly increased the SV. The NE-induced SV increases in responders were greater than that in non-responders (21.1 ± 5.4% versus 5.8 ± 5.5%, *P* < 0.001). Both Ea and Ees were increased by NE in both groups, and the increases in Ea were lower in responders (0.17 ± 0.22 mmHg/mL versus 0.39 ± 0.22 mmHg/mL, *P* = 0.008). However, the NE-induced increases of Ees in responders did not differ from that of non-responders (0.37 ± 0.26 mmHg/mL versus 0.32 ± 0.21 mmHg/mL, *P* = 0.577), thus Ea/Ees was normalized by NE in responders, while unchanged in non-responders (Table 3). The individual data on the Ea/Ees ratio for each patient is shown in Fig. 3.

NE also increased the SAP, DAP, and MAP in both groups. Besides, the HR was reduced by NE infusion in both groups. Accordingly, NE induced a significant increase in the T_tot-s_, but the T_pre-e_ was unchanged. Additionally, the administration of NE was associated with an increase in the LVEDV and VTI in both groups, but not the CVP. However, NE infusion resulted in a decrease of LVESV in responders, but not in non-responders. Thus, the LVEF and cardiac index were improved by NE in responders, yet not changed in non-responders (Table 3).

### Pearson correlation and logistic regression analysis

At baseline, the Ea/Ees ratio was positively correlated with the NE-induced SV increases (*r* = 0.688, *P* < 0.001), and was negatively correlated with the LVEF (*r* = − 0.809, *P* < 0.001) and SV (*r* = − 0.560, *P* = 0.001). The Ea/Ees ratio had no correlations with the HR, SAP, CVP, or LVEDV (all *P* > 0.05). In addition, the NE-induced changes in Ea seem to be more related to the changes in SAP (*r* = 0.802, *P* < 0.01) than that in SV (*r* = − 0.394, *P* = 0.021).

In the univariate logistic regression analysis, the baseline Ea/Ees ratio was identified as a potential predictor of SV response to NE (*P* = 0.009) (Table 4).

**Table 4:** Univariate logistic regression analysis for screening potential predictors of stroke volume response to norepinephrine

Variables	Odd ratio	95% CI for odd ratio		*P* value
		Lower	Upper	
HR (beats/min)	0.983	0.945	1.021	0.374
SAP (mmHg)	0.934	0.832	1.048	0.244
DAP (mmHg)	0.956	0.832	1.098	0.522
MAP (mmHg)	0.906	0.763	1.077	0.264
CVP (mmHg)	0.941	0.792	1.117	0.486
LVEDV (mL)	0.966	0.911	1.025	0.257
LVEF (%)	1.154	1.028	1.296	0.015
SV (mL)	1.042	0.976	1.114	0.218
VTI (cm)	1.302	1.032	1.643	0.026
NE dose (μg/kg/min)	1.778	0.469	6.738	0.397
Time from NE infusion start to MAP stabilization (min)	1.000	0.994	1.006	0.940
Ea/Ees ratio	0.008	0.000	0.293	0.009

SV stroke volume; NE norepinephrine; HR heart rate; SAP systolic arterial pressure; DAP diastolic arterial pressure; MAP mean arterial pressure; CVP central venous pressure; VTI velocity-time integral; LVEDV left ventricular end-diastolic volume; LVEF left ventricular ejection fraction; Ea effective arterial elastance; Ees left ventricular effective end-systolic elastance; CI confidence interval

### Receiver operating characteristic curve

**Fig. 4:**
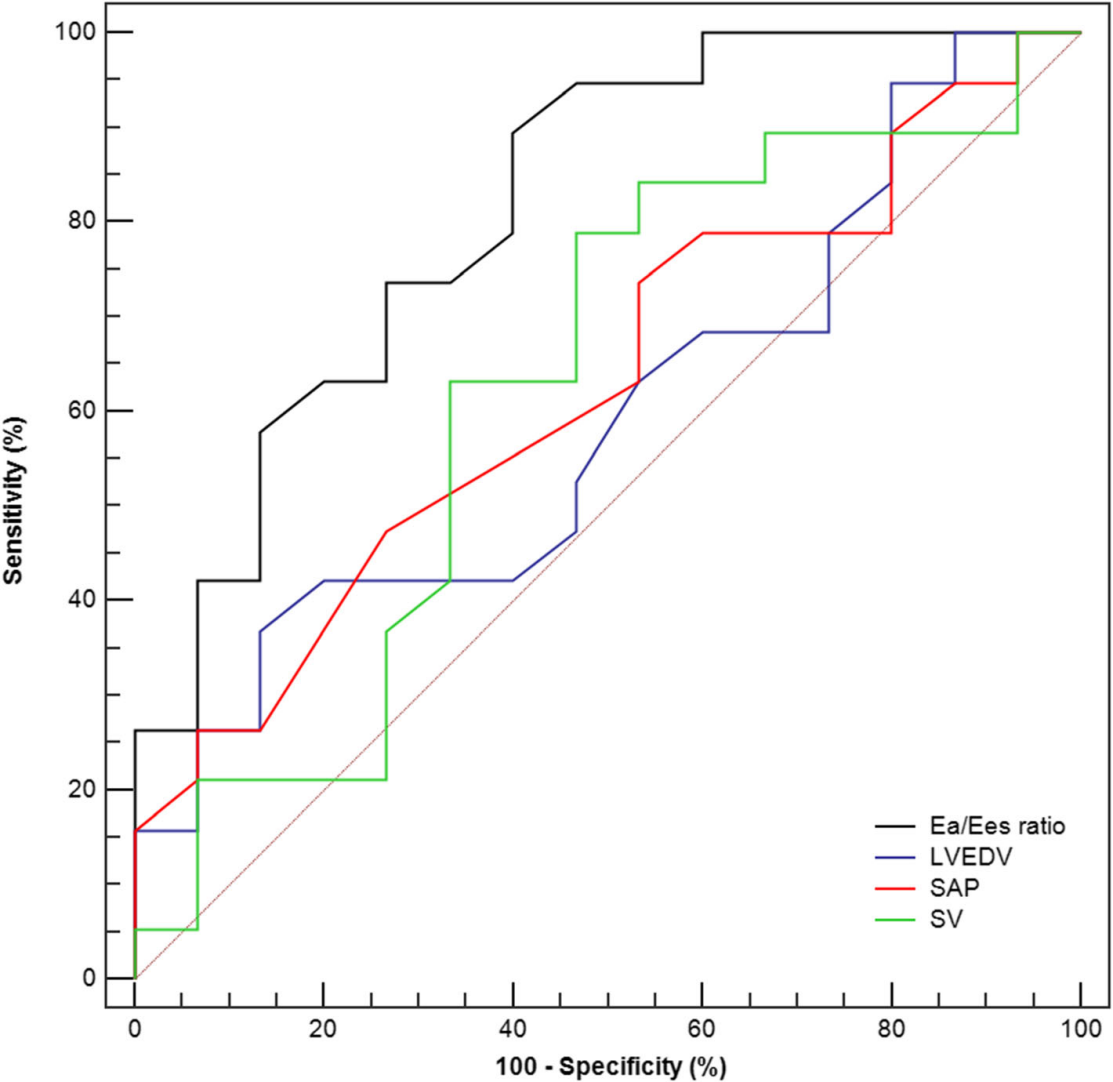
Receiver operating characteristic curves to discriminate stroke volume response to norepinephrine. Ea effective arterial elastance; Ees left ventricular effective end-systolic elastance; SV stroke volume; SAP systolic arterial pressure; LVEDV left ventricular end-diastolic volume

The ROC curves analyses suggested that the baseline Ea/Ees ratio could predict an increase ≥15% in SV after NE infusion, with an AUC of 0.816 (95% CI: 0.646 to 0.927, *P* < 0.001) (Fig. 4). The optimal cutoff value was 1.11, with a sensitivity of 89.5% (95% CI: 66.9 to 98.7%), a specificity of 60.0% (95% CI: 32.3 to 83.7%), a positive likelihood ratio of 2.24 (95% CI: 1.2 to 4.2), and a negative likelihood ratio of 0.18 (95% CI: 0.04 to 0.7). However, the baseline SV, SAP, and LVEDV had no ability to predict the SV response to NE, with an AUC of 0.626 (95% CI: 0.444 to 0.786, *P* = 0.218), 0.626 (95% CI: 0.444 to 0.786, *P* = 0.192), and 0.593 (95% CI: 0.412 to 0.758, *P* = 0.353), respectively.

## Discussion

This study was conducted to evaluate the predictive value of left VAC for the SV response to NE in septic shock patients. The results suggested that SV responders had an altered baseline left VAC, which was significantly greater than that in SV non-responders, and the baseline left VAC was positively correlated with the NE-induced SV increases. This study found that the baseline left VAC had the ability to predict SV response to NE infusion in septic shock patients, and the NE-induced SV increase was due to the normalization of left VAC, which was mainly attributed to the improvement of Ees rather than Ea.

Additionally, the current study suggested that both VTI and Ees were improved after NE infusion, indicating an improvement in cardiac contractility, which was consistent with the findings in a previous study [24]. However, the LVEF was not simultaneously improved in the non-responder group. This result is not surprising, because LVEF is not a reliable index of cardiac contractility, and it also depends on the ventricular afterload [23]. Several studies [25, 26] had demonstrated that fluid responsiveness was a factor that influenced the effects of various interventions on the left VAC. These studies [25, 26] found an increase in SV and a decrease in Ea, resulting in an improved left VAC, after fluid loading in fluid responders. Thus, confirmation of fluid non-responsiveness before NE infusion start was an important process in our study. Moreover, we did not allow the fluid challenge during the study period. Finally, the cumulative volume of fluid infusion during NE infusion was small, and it was similar between responders and non-responders. However, we found a significant increase in LVEDV in both groups. It could not conclude that NE increased the ventricular preload, because the small changes in LVEDV were probably not clinically relevant. Thus, the small fluid volume administered during NE infusion should have, if have to be considered, a very limited impact on our results.

Given that the changes in Ea and Ees were largely different between SV responders and non-responders, we speculated that the SV responsiveness to NE might be determined by the comprehensive effects of NE on the left VAC. In our study, we found that SV non-responders had a normal left VAC, Ea, and Ees at baseline. Administration of NE induced a similar improvement in both Ea and Ees, resulting in an unchanged left VAC, thus the potential increase in SV might be counterbalanced by the NE-induced increase in Ea which means a proportional increase in the end-systolic pressure (ESP) at a given SV. On the contrary, SV responders had an abnormal baseline left VAC (Ea/Ees ratio > 1.36) that mainly resulted from impaired Ees. After NE administration, the left VAC was normalized, and it was mainly attributed to the improvement of Ees rather than Ea. The large improvement in Ees finally caused a significant increase in SV despite the small increase of Ea. These findings indicated that NE seemingly exerted a main inotropic effect in patients with abnormal left VAC, and exerted similar inotropic and vasoconstrictive effects in patients with normal left VAC. Furthermore, the comprehensive effect of NE on the interaction between cardiac and arterial performance was determined by the baseline left VAC. Our study suggested the ability of the baseline left VAC to predict the SV response to NE infusion, which was consistent with the result from the study by Guinot et al. [19]. Differently, the study by Guinot et al. [19] recruited post-cardiac surgery patients who usually have low CO and high peripheral vascular resistance, which is different from the hemodynamic profile of septic shock that generalized vasodilation resulting in high CO with or without myocardial depression.

Over past decades, Ea has been widely recognized as a measure of ventricular afterload [20, 27]. According to the calculation formula, Ea is the change in ESP for a given change in SV, and it reflects all the extracardiac forces opposing to ventricular ejection [27]. Of note, a previous study [26] found a poor correlation between fluid-induced changes in Ea and those in ESP (ESP was estimated as 0.9 × SAP), and concluded that Ea should not be used in isolation as an index of left ventricular afterload. Inconsistent with the previous study, the current one indicated that the NE-induced increases in Ea were related well to the NE-induced increases in SAP (*r* = 0.802, *P* < 0.01). Different interventions might be a potential explanation for these conflicting findings. In their study, fluid loading primarily increased the SV and thus led to a reduction of Ea, despite the increases in SAP. Conversely, in our study, NE increased the Ea by improving the SAP through its main vasoconstrictive effect. Based on these findings, whether Ea can be considered as an index of ventricular afterload still needs more discussion.

The current study has a main clinical implication that the evaluation of left VAC before NE infusion is helpful to identify which population will benefit from the use of NE. Maintenance of perfusion pressure while still sustaining adequate CO is crucial for hypotensive patients [9]. Theoretically, among hypotensive patients treated with NE to restore the arterial pressure, those patients with increased SV after NE infusion may suffer from better clinical prognosis than those with unchanged or decreased SV. Our study indicates that septic shock patients with a baseline left VAC > 1.11 are more likely to improve the SV with use of NE. For those septic shock patients with a baseline left VAC < 1.11, the abuse of a large dose of NE should be avoided because its great afterload effects on the left ventricle might reduce the SV. Accordingly, our study provides a new perspective that dynamic assessment of left VAC during the resuscitation of septic shock may be a promising monitoring strategy to guide titrated adjustment of NE dosage to optimize the cardiac work efficacy and thus improve clinical prognosis.

There are several limitations to our study. Firstly, the small fluid volume administered during the study period might affect the left VAC to a small extent. While we had restricted changes in some variables that might affect the left VAC (e.g. IMV setting, fluid challenge), the fluid administration was not completely restricted during the study period because it was unrealistic in the clinical practice due to the relatively long study period (median duration of 95 min). Secondly, IMV and sedative and analgesic drugs may also be confounders affecting the left VAC due to its hemodynamic effects [28–30]. Unfortunately, we did not analyze the IMV parameters and the dose of sedatives and analgesics because of the limited sample size. Nevertheless, the use of IMV and sedatives or analgesics would not prevent the deduction of the conclusion, because modifications of these variables were not allowed during the study period.

Lastly, as discussed previously [19, 23, 25], the method used for the estimation of Ea and Ees remains a challenge for the reliability of our findings. Estimation of Ees in our study was based on the noninvasive single-beat method [21] that assumed a load-independent linear end-systolic pressure-volume relationship and a constant volume axis intercept (V0) of the relationship curve. However, a previous study reported a significant correlation between the V0 and cardiac function [14]. Thus, changes in V0 resulted from impaired cardiac contractility might affect the estimation of Ees. Furthermore, we measured the radial arterial pressure as a surrogate of aortic systolic pressure to calculate the Ea. However, the radial arterial pressure was reported to be less accurate than the femoral arterial pressure to estimate the Ea [26] and it may be imprecise to represent the aortic systolic pressure in septic shock due to the collapsed circulatory system [23]. Even so, it would not affect the precision of calculation of Ea/Ees ratio because of the similar influences on the calculation of Ea and Ees. Thus, the left VAC can be considered as a valid predictor of SV response to NE.

## Conclusions

Administration of NE induced changes in Ea and Ees in patients with septic shock. The SV response to NE was determined by the comprehensive effects of norepinephrine on the left VAC, which depended on the left VAC at baseline. The baseline left VAC had predictive value for the SV response to NE infusion in patients with septic shock.

## Data Availability

The datasets used and/or analyzed during the current study are available from the corresponding author on reasonable request.

## Abbreviations

ICU: intensive care unit
NE: norepinephrine
CO: cardiac output
DAP: diastolic arterial pressure
SV: stroke volume
VAC: ventricular-arterial coupling
Ea: effective arterial elastance
Ees: left ventricular effective end-systolic elastance
CVP: central venous pressure
LVEF: left ventricular ejection fraction
MAP: mean arterial pressure
TTE: transthoracic echocardiography
IMV: invasive mechanical ventilation
APACHE: acute physiology and chronic health evaluation
SOFA: sequential organ failure assessment
PaO2: arterial oxygen partial pressure
FiO2: fractional inspired oxygen
LVEDV: left ventricular end-diastolic volume
LVESV: left ventricular end-systolic volume
VTI: aortic velocity-time integral
T_pre-e_: pre-ejection time
T_tot-s_: total systolic time
LVOT: left ventricular outflow tract
HR: heart rate
SAP: systolic arterial pressure
ESP: end systolic pressure
SD: standard deviation
IQR: interquartile range
ROC: receiver operating characteristic
AUC: area under the ROC curve
CV: coefficient of variation
